# Neural Activation During Submaximal Contractions Seems More Reflective of Neuromuscular Ageing than Maximal Voluntary Activation

**DOI:** 10.3389/fnagi.2016.00019

**Published:** 2016-02-25

**Authors:** Gil Scaglioni, Marco V. Narici, Alain Martin

**Affiliations:** ^1^Université de Bourgogne Franche-Comté (UBFC), Cognition Action et Plasticité Sensorimotrice (CAPS) UMR1093Dijon, France; ^2^Institut National de la Santé et de la Recherche Médicale (INSERM U1093), Cognition Action et Plasticité Sensorimotrice (CAPS) UMR1093Dijon, France; ^3^School of Graduate Entry to Medicine and Health, Faculty of Medicine, MRC-Arthritis Research UK Centre of Excellence for Musculoskeletal Ageing Research, University of NottinghamDerby, UK

**Keywords:** twitch interpolation, voluntary contraction, voluntary activation, triceps surae, ageing

## Abstract

This study aimed at testing the hypothesis that differences in neural activation strategy during submaximal but not maximal plantarflexions exist between young and older men. Eleven young men (YM, 26 ± 4 years) and thirteen old men (OM, 76 ± 3 years) volunteered for the investigation. Maximal voluntary torque (MVT) was 38.2%, lower (*p* < 0.001) in OM than in YM, while voluntary activation was equivalent (~97%). The relationship between the interpolated twitch-torque and the voluntary torque (IT-VT relationship) was composite (curvilinear + exponential) for both age-groups. However, the OM showed accentuated concavity, as attested by the occurrence of the deviation from linearity at a lower contraction intensity (OM: 54.9 vs. YM: 71.9% MVT). In conclusion, ageing does not affect the capacity to fully activate the plantar flexors during maximal performances, but it alters the activation pattern for submaximal levels of effort. The greater age-related concavity of the IT-VT relationship suggests that, during submaximal contractions, OM need to reach a level of activation higher than YM to develop an equivalent relative torque.

## Introduction

Old age is typically characterized by a decrease in the force-generating capacity of human skeletal muscles. This decline usually exceeds the force loss predicted by muscle atrophy leading to a decrease in force per muscle unit area (Jubrias et al., 1997; Delbono, 2002; D’Antona et al., 2003; Morse et al., 2005). This dissociation suggests that factors other than muscle atrophy may contribute to muscle weakness. Indeed, as recently reported by Moore et al. (2014) in individuals aged 85 + the loss of force is about fourfold greater than the decrease in muscle size. Apart from qualitative changes in contractile properties (Hunter et al., 1999; Delbono, 2002; Goodpaster et al., 2006) and muscle composition (Andersen et al., 1999; D’Antona et al., 2003), this loss of strength, greater than that expected on the basis of morphological changes, might depend on the inadequate activation of the available muscle mass by the voluntary neural drive (Kamen et al., 1995; Enoka et al., 2003; Kido et al., 2004). The level of voluntary activation (VA) is a measure that represents the nervous systems overall capacity to fully activate skeletal muscle (Allen et al., 1995; Taylor, 2009), and is generally quantitated by electrically stimulating the motor nerve to a muscle during a maximum voluntary contraction (MVC).

The literature has so far associated age-related alteration in voluntary neural drive with a deficit detected during maximal efforts. Although conflicting evidence still exists regarding the ability of older adults to fully activate skeletal muscle, most of studies reported preserved functionality of the descending motor pathways with ageing, for the upper as well as for the lower extremities (for a review, see Klass et al., 2007).

Despite the reports of a full activation capacity with old age, some studies pointed out differences between the young and the older in the neural behavior (i.e., altered balance between excitation and inhibition of spinal circuits, increased coactivation of agonist and antagonist muscles, an earlier shift from recruitment vs. rate coding motoneurons activation strategy) adopted to maintain motor output at equal levels during submaximal contractions (Earles et al., 2001; Akataki et al., 2002; Baudry et al., 2010). Accordingly, confining investigations solely to the maximal capacities of the neuromuscular system could limit our understanding of the mechanisms involved in neural activation at muscle contraction levels observed in everyday life.

Hence, linking the relationship between muscle VA with contraction intensity seems particularly appropriate. This approach has the advantage of enabling one to identify a muscle activation “pattern” ranging from weak efforts to maximal performances and understand the underlying motor unit activation strategies in the older. In an earlier investigation, focused on the triceps surae, we developed a model describing the relationship between interpolated twitch torque (IT, plotted as *y*-axis) and voluntary torque (VT, plotted as *x*-axis; Scaglioni et al., 2002). The main interest of this model is that it provides three relevant parameters: (i) the maximal expected torque (MT_exp_), i.e., the torque that the subject would be able to produce for complete motor units activation (when VA = 100%; intercept on *x*-axis); (ii) the expected resting twitch torque (RT_exp_), i.e., the index of the muscle “true” mechanical contractile properties (intercept on *y*-axis); and (iii) since the model depicts composite behavior (with a linear section followed by a curvilinear portion), it also permits localization of a deviation point (DP), namely the contraction intensity at the transition between the two components. This parameter likely reflects the level of voluntary effort at which a modification of the electromechanical efficiency (i.e., ratio between neural activation and motor output of the muscle) of the plantarflexion muscles occurs (Scaglioni et al., 2002).

Even though the model was first developed for elderly adults and then validated for young individuals (Scaglioni et al., 2002; Scaglioni and Martin, 2009), it remains unclear whether the activation strategy of plantar flexors (PFs), from weaker to maximal efforts, is affected by ageing. Thus, the aim of this study was to investigate, in young and older individuals, differences in neural activation pattern reflecting an age-related alteration of the strategies used to modulate and maintain motor output over a wide range of force levels. In particular changes in neuromuscular behavior at low force levels are particularly relevant from a functional point of view since submaximal contractions more closely resemble functional daily tasks, the decline of which may profoundly impact one’s ability to live independently. Since muscle mass and composition are remodeled with age, and since an ongoing shift of motoneuron control seems to occur from spinal to cortical mechanisms (Butchart et al., 1993; Earles et al., 2001; Baudry et al., 2010; Klass et al., 2011), one could expect an overall change in the IT-VT relationship signaling a modified activation pattern.

## Materials and Methods

### Subjects

Eleven healthy young men (YM, age: 26 ± 4 years, mass: 72 ± 7 kg, height: 1.78 ± 0.05 m) and thirteen healthy older men (OM) living independently (age: 76 ± 3 years, mass: 73 ± 10 kg, height: 1.68 ± 0.04 m) gave their written consent to participate in this study after being fully informed of the experimental procedures. The older subjects were all medically screened before taking part in the investigation. The medical criteria applied for the exclusion of subjects were: presence of known neurological, musculoskeletal, metabolic and inflammatory disorders, systemic cardiovascular and neoplastic diseases. The study was approved by the University of Burgundy Committee on Human Research and all experimental procedures were performed in accordance with the Declaration of Helsinki.

### Plantar Flexors Mechanical Output: Experimental Set-Up

The PFs isometric maximal voluntary torque (MVT) and the evoked twitch contraction were performed in the sitting position with the trunk inclined at 30° to the vertical. All measurements were carried out on the dominant lower limb, determined by pushing the back of the subject as hard as needed to force him to take a step forward, the leg he stepped forward with being considered as dominant. The dominant limb was in a standardized position of 90° flexion at the hip, knee and ankle joints. Measurements were assessed using a custom-made pedal equipped with strain gauges (Société Doerler-Vandoeuvre, France) and an amplifier (PM Instrumentation, model 1300B, amplification: 8 *V* = 200 Nm), specifically developed for the study by the mechanics workshop of the local engineering school (I.U.T. Génie Mécanique, Dijon, France). A strap was placed around the foot to secure it firmly to the pedal and thus create an effective mechanical constriction. The amplified mechanical signal was digitized on-line by a 16-bit analog-to-digital converter (ITC-16 Computer Interface, Instrutech Corporation, Great Neck, NY, USA). Torque was sampled at a frequency of 2 kHz and stored for analysis with commercially available software (Tida, Heka Elektronik, Lambrecht/Pfalz, Germany).

### Nerve Stimulation: Experimental Set-Up

In order to evaluate the level of activation, during isometric efforts, an electrical stimulator (DS7, Digitimer Ltd, Welwyn Garden City, UK), with a constant-current (400 V) and adjustable intensity, was used to percutaneously deliver square-wave stimuli of 1 ms in duration. Single pulses were delivered to the posterior tibial nerve via a cathode ball electrode (0.5 cm Ø) manually pressed onto the popliteal fossa and a large anode electrode (12.5 cm × 7.5 cm, VersaStim, ConMed, NY, USA) placed on the anterior surface of the knee. Even though other methods are proposed in the literature (Flynn et al., 2012), we chose to stimulate the nerve trunk by single pulse since: (a) this methodology is less uncomfortable for a frail population; and (b) we previously showed that the stimulation site (nerve trunk vs. muscle) and the number of pulses (single vs. double pulse) do not affect the assessment of muscle activation during maximal and sub-maximal voluntary contractions (Scaglioni and Martin, 2009). Moreover, during submaximal contractions the nerve trunk stimulation method demonstrates even a greater consistency by ensuring the recruitment of a similar proportion of the motor pool irrespective of the number of pulses (Scaglioni and Martin, 2009).

Subjects were familiarized with sub-maximal electrical stimuli over a period of 10 min prior to the testing session. The current amplitude was determined by progressively increasing the amperage until maximal twitch torque and maximal soleus M wave were attained. For this reason, pairs of silver-chloride surface electrodes 8 mm in Ø (2 cm interelectrode distance) were placed along the mid-dorsal line of the leg at ~4 cm below the distal insertion of the gastrocnemii into the Achilles tendon. The reference electrode was placed in a central position between the gastrocnemii bellies. Electromyographic (EMG) signals were amplified with a bandwidth frequency ranging from 15 Hz to 5 kHz (common mode rejection ratio = 90 dB; impedance = 100 MΩ, gain = 1000). The EMG signal was digitized on-line (sampling frequency: 10 kHz). The maximal stimulation amplitude was further amplified by 10% to ensure the use of a supramaximal-current stimulus. The supramaximal amplitude ranged from 50–86 mA for the YM and from 44–140 mA for the OM.

### Experimental Protocol

Subjects performed three 5 s long MVC of PFs. Throughout these attempts to perform maximal effort, the subjects were given precise instructions and standardized verbal encouragement (cf. Gandevia, 2001). The electrical stimulation was delivered 3 s before each MVC to determine the resting twitch (RT) and when the VT reached its plateau (IT). Subsequently, the subjects performed, in randomized order, voluntary contractions at eight target levels: 20, 30, 40, 50, 60, 70, 80, and 90% of their MVT. The target efforts and the exerted torque were displayed, in real time, on the computer monitor as visual feedback. Once the performed torque was considered to match the reference in a stable way, the electrical stimulation was delivered to the contracting muscle. To avoid any interference due to fatigue, subjects were given a 2 min rest between trials.

### Data Analysis

#### MVT, Muscle Activation, MT Expected, RT Expected, Deviation Point

Only the highest MVT and the corresponding level of VA were considered for analysis. It was observed that the RT_exp_ is equivalent to the post-contraction RT (i.e., the twitch torque evoked after the maximal effort) and may thus replace it for calculating VA (Scaglioni and Martin, 2009). However, to verify whether, in OM as in YM there is no difference in predicting the MVT of the PFs with potentiated and unpotentiated stimuli (Behm et al., 1996) the VA of the PFs was calculated twice according to the equation:

(1) VA% = (1-IT/resting​ twitch torque×100)

with two different resting twitch factors: RT, RT_exp_.

In fact, it seems noteworthy that the potentiation equivalence for the IT and the post-contraction RT has not been fully tested. The post-contraction RT is usually evoked a few seconds after the effort, but since the decay of potentiation is exponential after the maximal voluntary contraction and may be different between populations, caution should be used when assuming that the degree of potentiation is equivalent for the IT and the post-contraction RT.

Measurements of the interpolated twitch amplitude obtained at the target levels of effort (20–100% MVT) enabled us to plot the relation between IT and VT. Especially for the higher contraction levels (>60% MVT), the torque developed by the subjects did not always precisely match the target line, but the off-line analysis allowed precise determination of the torque value at which the stimulus was triggered. The mechanical IT seems to decrease in a non-linear fashion as contraction intensity increases, showing for each subject a linear part and a curvilinear portion. The model (see *Eq. 2*) proposed in our previous study (Scaglioni et al., 2002) depicts this composite behavior and satisfies the requirements for assessing the relevant interception points between curve and axes, namely RT_exp_ at VT = 0, and MT_exp_ at IT = 0.

(2) $$VT\;=\;a\left[(1-b)\;e^{cIT}+b\left(1-\frac{IT}{d}\right)\right]$$

Coefficient “*a”* corresponds to MT_exp_, while “*d”* corresponds to RT_exp_. Linear and exponential components are weighted by coefficient “*b”*, while “*c”* is the constant of the exponential portion. The four coefficients were estimated for each subject by feeding a nonlinear regression software package (SYSTAT 5.2.1 for Macintosh, Inc., USA) with individual (IT, VT) data. It is worth mentioning that the descriptive ability of the chosen function incorporates both curvilinear and purely linear relationships; in the latter case, coefficients *b* and *c* are respectively equal to 1 and 0.

Furthermore, the model enabled determination of the DP as the contraction intensity at which the relation changed from linear to curvilinear. For this purpose, a threshold in the curve slope was introduced as parameter *k*. This process was formalized in a previous study (Scaglioni et al., 2002; for details, see “Materials and Methods” Section).

#### Statistical Analysis

The data are presented as means ± *SD*. The criteria of normality were tested using the Shapiro-Wilks’ W test. A two-way ANOVA with repeated measures on the 2nd factor examined the effects of the *group* (YM vs. OM), the effects of the *assessment criterion* (expected vs. actual performance) and their interactions on the maximal torque and on the rest twitch torque measures. A second two-way ANOVA with repeated measures on the 2nd factor examined the effects of the *group* (YM vs. OM), the *rest twitch torque type* (RT vs. RT_exp_) and their interactions on the estimation of VA. When a major effect or an interaction was found, a *post hoc* analysis was computed using the Scheffe’ test. Linear regression analysis (Pearson product–moment correlation) was used to determine the degree of association between MVT (expressed as a percentage of MT_exp_) and VA%. The best degree of association between IT and the voluntary isometric effort was determined by applying linear regression analysis and a non-linear correlation test. The difference between the linear and the non-linear correlation coefficients was determined by applying a Student’s paired *t*-test. The one-sample Student’s *t*-test was used to evaluate the difference between the VA and 100%. The age effect was determined applying a Student’s paired *t*-test unless the data did not meet the criteria of normality, in which case a nonparametric Mann-Whitney U test was applied. Statistical analysis was performed using Statistica (Statsoft, 10 version, Statistica, Tulsa, OK and software package SYSTAT 5.2.1, Inc., USA). The critical level for statistical significance was set at 5%.

## Results

### Maximum PFs Torque and Twitch Torque

**Figure 1:**
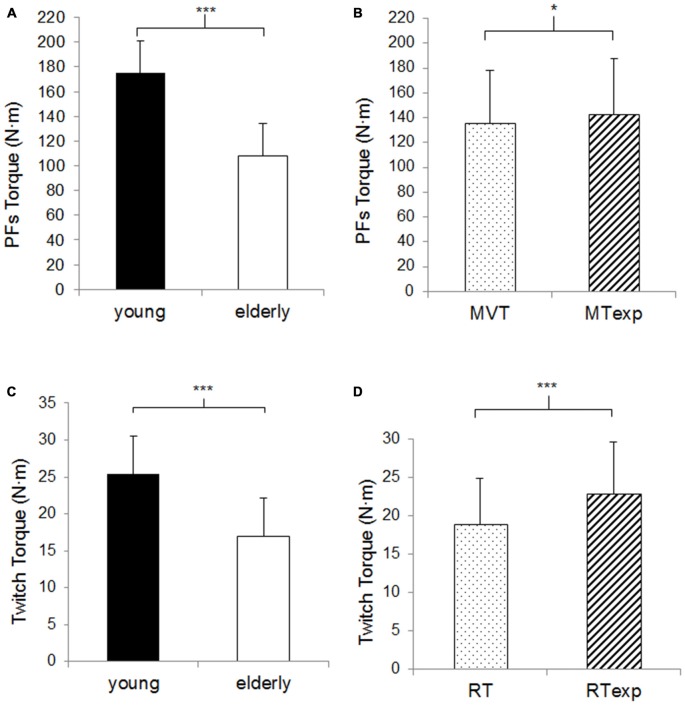
**Plantar flexors (PFs) maximal torque and twitch torque. (A)** The columns correspond to the average values of the PFs maximal torque in the young (*n* = 11, closed columns) vs. elderly population (*n* = 13, open columns). **(B)** The columns correspond to the average values of the actual (dotted columns) vs. expected (hatched columns) PFs maximal torque (MVT vs. MT_exp_). **(C)** The columns correspond to the average values of the twitch torque in the young vs. elderly population. **(D)** The columns correspond to the average values of the actual vs. expected rest twitch torque (RT vs. RT_exp_). Data are presented as mean values ± *SD* for the expected and actual measure and for the young and elderly subjects, given that the ANOVA analysis did not reveal any assessment criteria or age effect. *Significant difference *p* < 0.05. ***Significant difference *p* < 0.001.

There was a significant age effect for the maximal PFs torque, 38.2% lower in the OM compared to the YM (*p* < 0.001, Figure 1A), and a significant effect of the assessment criterion, in fact despite the great data dispersion, the MVT was slightly lower than the MT_exp_ (4.3 ± 7.2%, *p* < 0.01, Figure 1B), but no significant interaction was observed.

There was a significant age effect for the rest twitch torque, which was lower in OM compared to the YM by a similar percentage as the maximal torque production (33.0%, *p* < 0.001, Figure 1C) and there was a significant effect of the assessment criterion, the RT was 17.4 ± 10.6% lower than the RT_exp_ (*p* < 0.001, Figure 1D), but no significant interaction was observed.

Since, as previously mentioned, the descriptive ability of the composite model also incorporates purely linear relationships, it was found that one senior subject presented a better linear adjustment, thus for this subject the MT_exp_ and the RT_exp_ were linearly extrapolated.

### Voluntary Activation

Since the actual RT was significantly lower than the RT_exp_ (see above) a *rest twitch torque type* factor (RT vs. RT_exp_) was added to the ANOVA analysis in order to determine its impact on the VA assessment. No *age* or *rest twitch torque type* effect was observed. The VA ranged from 96.8 ± 3.3% to 97.4 ± 2.6%.

The slight deficit in VA (100%–VA% in YM: 2.7 ± 2.7% *p* < 0.01 vs. OM: 2.9 ± 3.0% *p* < 0.01) was similar in YM and OM and close to the difference between MVT and the MT_exp_ in the two age-groups (YM: 4.3 ± 5.8% vs. OM: 4.3 ± 8.4%).

In a previous study conducted on YM (Scaglioni and Martin, 2009) we observed that the MVT expressed as a percentage of the MT_exp_ changed *pari passu* with the level of VA. Indeed, a linear correlation was observed between MVT (%MT_exp_) and VA% values (*r* = 0.95, *p* < 0.01), suggesting that these two parameters provide similar outcomes. It is interesting to observe that this correlation persists into old age (*r* = 0.68, *p* < 0.01). Taken together, these results allow us to conclude that maximal activation capacity of the PFs is preserved into old age.

### IT-VT Relationship

The present results corroborate the non-linearity of the IT-VT relationship for PFs, in YM and OM. Indeed, it was observed that the composite regression, described by *Eq. 2*, fitted the experimental points better as compared to the linear adjustment in YM (*R*^2^ non-linear = 0.96 ± 0.02 > *R*^2^ linear = 0.92 ± 0.03, *p* < 0.001) as well as in OM (R^2^ non-linear = 0.93 ± 0.06 > *R*^2^ linear = 0.87 ± 0.09, *p* < 0.01).

**Figure 2:**
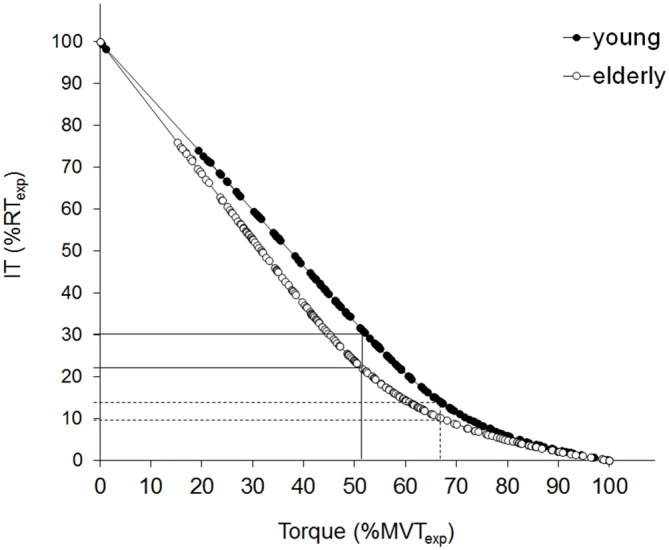
**Relationship between the interpolated twitch torque (IT), and voluntary isometric torque.** Superimposed twitch torque (IT), evoked by supramaximal stimulus intensity, is plotted against the corresponding contraction level, for the young (filled circles) and the elderly (open circles) population. The composite regressions are: *Torque = 179.0 [(1 − 0.745) e^−0.583IT^ + 0.745 (1 − IT/27.3)]* for the young cluster (*n* = 12) vs. *Torque = 113.8 [(1 − 0.632) e^−0.665IT^> + 0.632 (1 − IT/19.3)]* for the elderly group (*n* = 11). The specific equations are obtained by substituting the mean coefficients values derived from the individual fitting into *Eq.2*. Torque values are then expressed as a % of the expected maximal voluntary torque (MVT_exp_), while IT is expressed as a % of the expected twitch torque at rest (RT_exp_). The composite regression function provides an estimation of the point of deviation from linearity. The full lines indicate the MVT_exp_% corresponding to the seniors’ deviation point (DP) and the relative RT_exp_% for both populations. The dotted lines indicate the MVT_exp_% corresponding to the young *DP* and the relative RT_exp_% for both populations.

As depicted in Figure 2, a comprehensive non-linear regression for each population may be obtained by directly substituting the mean coefficients values (i.e., *a, b, c, d*) obtained for each group from the individual fittings into *Eq. 2*. Coefficients “*a”* and “*d”* correspond, respectively, to MT_exp_ and RT_exp_ (see previous sections for details). Coefficient “*b”* was significantly lower (*p* < 0.05) for the older population while coefficient “*c”* was slightly higher, but this difference failed to be statistically significant, possibly because of the great data dispersion exhibited by both populations (see Table 1).

**Table 1:** **Age effect on the deviation point and coefficients of the equation: VT = a [(1 - b) e^cIT^ + b (1 - IT/d)]**.

	Young Men	Elderly Men
DP (Nm)	122.4 ± 18.8	58.3 ± 16.9***
a or MT_exp_ (Nm)	179.0 ± 26.8	113.8 ± 29.2***
b	0.745 ± 0.067	0.632 ± 0.110*
c	−0.583 ± 0.456	−0.665 ± 0.398
d or RT_exp_ (Nm)	27.3 ± 4.9	19.3 ± 5.4**

Values are means ± SD. Young men n = 11, elderly men n = 12 (for 1 elderly men the IT-VT relation was best matched by a linear regression). DP: is the deviation point, a or MT_exp_: is an estimation of the maximal voluntary torque, b: weights the linear and the exponential components of the equation, c: is the constant of the exponential portion, d or RT_exp_: is an estimation of the resting twitch torque. Young vs. elderly is statistically different, *p < 0.05; **p < 0.01; ***p < 0.001.

The specific equations thus attained are:

(3) $$\begin{array}{l} VT\;=\;179.0\;[(1-0.745)e^{\;-\;0.583IT}+0.745\;(1-IT/27.3)]\text{​​​​ }\\ \text{​ for the young subjects} \end{array}$$

(4) $$\begin{array}{l} VT\;=\;113.8\;[(1-0.632)e^{\;-\;0.665IT}+0.632\;(1\;-\;IT/19.3)]\\ \text{ for the older individuals} \end{array}$$

The VT was derived by replacing the measured IT values in each equation.

As mentioned in the “Data Analysis” section, the composite regression function may provide an estimation of the point of deviation from linearity. The mean value for each population may be expressed (i) as an average of the individual *DP* values (the mean value in the older group was calculated on 12 subjects since one presented a better linear adjustment); or (ii) it may be derived from the comprehensive fitting equations (*Eq. 3 and 4*). The two approaches gave equivalent results (68.8 ± 8.5% and 68.2% for YM vs. 52.4 ± 13.3% and 52.3% for OM, values are expressed as a percentage of the MT_exp_) and demonstrated a significant difference between the young and the older subjects (*p* < 0.01). The more pronounced steepness (i.e., b/d) of the linear component of *Eq.2* (0.028 ± 0.003 for YM vs. 0.035 ± 0.012 for OM, *p* < 0.05) associated with the earlier deviation from linearity shown by the OM is responsible for a more marked concavity in their IT-VT relationship. This indicates that, for efforts ranging from ~20–80% of the MT_exp_, the corresponding IT is lower for the seniors compared to the young individuals.

## Discussion

The main finding of this study is that, even though ageing does not affect the ability of healthy adults to fully activate the PFs during maximal voluntary contractions, it alters the activation strategy adopted to produce submaximal efforts, as required by most activities of daily living.

The two approaches employed to evaluate the VA yielded the same conclusion regarding the ability of older subjects to maximally activate the PFs. The first, which currently represents “the gold standard” for assessing maximal VA, involved measuring the VA during MVC (Allen et al., 1995). With this approach, it was found that maximal VA of PFs was slightly but significantly lower (i.e., ~3%) than 100% in both groups of subjects. This result was confirmed by the multiple-point extrapolation method which provides an estimation of the real maximal torque, i.e., the torque that the subject would be able to produce for a total IT occlusion. In effect, the difference between the MVT and the MT_exp_ could be viewed as an alternative approach to quantify the muscle activation deficit (De Serres and Enoka, 1998). In other words, the higher value of the expected torque vs. the actual maximal torque, observed in the present study for both populations, indicates that there is a gap between what can be performed voluntarily and what may be achieved with total twitch occlusion. This deficit, equivalent again in the two age-groups (~4%) and close to the deficit in VA%, highlights once more a preservation of central neural drive with ageing. This method is useful not only because it provides an alternative multiple-points procedure for estimating the capacity to fully activate a muscle, but also because it enables the use of submaximal rather than maximal efforts.

Had the present analysis been restricted to conditions of maximal voluntary capacity, the inference would have been made that, in the older population, the integrity of the descending neural drive is accompanied by a significant deficit in maximal voluntary strength (~−38%). Thus the conclusion would have been made that the age-related decrease in force production basically results from alterations within the muscle and its nerve supply. The age-related deficit in the RT torque (expected or actual), the extent of which is similar to that in MVT, supports this result. Indeed, key factors usually responsible for lower RT values in old age, are: (i) changes in muscle composition, with a selective atrophy or loss of fast-twitch motor units (Doherty et al., 1993; Lexell, 1995; Verdijk et al., 2007; Nilwik et al., 2013) and an increase in the relative number of motor units with slow and transitional properties (Doherty et al., 1993; Luff, 1998; Andersen et al., 1999; Andersen, 2003; D’Antona et al., 2003); (ii) an increased proportion of non-contractile material within the muscle (Hasson et al., 2011); (iii) alterations in the excitation-contraction coupling process (Payne and Delbono, 2004; Boncompagni et al., 2006; Delbono, 2011); and (iv) increase in tendon compliance (Narici et al., 2008; Stenroth et al., 2012).

However, since this study investigated the entire relationship linking muscle activation to contraction intensity, the data analysis could be extended to submaximal conditions, enabling to make novel observations on the impact of ageing on muscular efforts of intensity similar to that of daily activities. First of all, it was observed that the relationship between the extra torque produced by the interpolated twitch and the level of voluntary isometric torque was nonlinear for both populations studied, suggesting that the curvilinear shape of the IT-VT relationship is unrelated to age, hence muscle-dependent. However, the noteworthy result is that the concavity of the IT-VT relationship is more pronounced for the older population (Figure 2). This observation is evidenced by the earlier departure from linearity of the relationship (with a mean DP at ~52% of the MVT_exp_ for OM and at ~68% for younger subjects) but also by the greater steepness of its linear part, as compared with that in the younger population. Thus, from a functional point of view, the impact of ageing on the concavity of the IT-VT relationship implies that, for sub-maximal levels of efforts (between ~20 and 80% MVT, see Figure 2), older subjects need to reach higher levels of activation than their younger counterparts to develop an equivalent relative torque. Older individuals have to activate more MUs than younger subjects to generate force by a similar percentage. Since the axes of the relationship are normalized (i.e., torque normalized by MVT_exp_ and IT normalized by RT_exp_) the difference between younger and older subjects should be attributed to parameters other than changes in peripheral muscle structure. While it is impossible to exclude factors such as an earlier contribution of the unstimulated synergist muscles, the shape alteration of the IT-VT relationship indicates a modification of the neural strategies adopted to produce submaximal muscle efforts. The smoothing of the relationship at lower levels of effort with ageing could therefore be due to earlier modification of the motor unit activation pattern (recruitment vs. rate coding) or activation strategy among synergistic muscles. It is widely documented that, with ageing, motor units undergo substantial remodeling resulting in a decrease in fiber size and number, but an increase in fiber number per motor unit (Deschenes, 2011) and a slowdown of contractile properties (Doherty et al., 1993; Lexell, 1995; Verdijk et al., 2007; Nilwik et al., 2013). The earlier departure from linearity of the IT-VT relationship with ageing may represent an earlier change in the neural strategy. One may in fact hypothesize that these remodeled motor units are fully activated in muscle efforts significantly lower than in younger individuals, possibly due to increased homogeneity of axonal diameters (Scaglioni et al., 2003) and that the further increase in muscle force merely depends on increased motor unit rate coding. This finding suggests that the typical increase in the innervation ratio of motor units due to sprouting, is not effective enough to counterbalance age-related muscle weakening, due to the loss of motor units (Scaglioni et al., 2003). Consequently, the “neural cost” of contractions performed in daily activities would be expected to be greater in old age. Indeed, OM are close to their maximal activation capacity (~80%) for efforts around 50% of their maximal strength capacity, possibly to counteract, *inter alia*, excessive antagonist coactivation (for review, see Hortobágyi and Devita, 2006).

The smoothing of the relationship at lower levels of effort in OM could also be dependent on a modified synergism between agonist and antagonist muscles. A number of studies have found that, when performing steady contractions, there can be differences between young and old adults in the amplitude of force fluctuations about an average value (for a review, see Enoka et al., 2003), and that this difference is greatest at low forces. One of the factors most responsible for force fluctuation is the coactivation of the antagonist muscles. Moreover, previous studies have shown that young and older individuals adopt different neural strategies to maintain motor output at equal levels during PFs and dorsiflexors submaximal contractions. Young subjects modulate presynaptic inhibition, whereas older subjects rely less on presynaptic inhibition and more on direct motor activation (Butchart et al., 1993; Earles et al., 2001; Klass et al., 2011). Thus, the lack of modulation in presynaptic inhibition with contraction in OM may indicate a shift to cortical mechanisms for motor control. All together these results may further account for the earlier departure from linearity of the IT-VT relationship in older individuals.

In summary, even though the capacity to fully activate a muscle group crucial for the maintenance of standing balance is preserved with ageing, an altered activation strategy is observed for submaximal levels of effort. It is thus clearly restrictive to confine analysis to maximal efforts if the purpose is to address age-related alterations in the voluntary neural drive. Indeed, maximal activation capacity does not appear to be a sufficiently sensitive criterion to investigate neuromuscular ageing, especially given that most daily activities are performed at submaximal intensities, and that with ageing a more sedentary lifestyle is usually adopted leading to fewer moderate-effort contractions. The mere analysis of the two extremes of the IT-VT relationship (i.e., IT and MVT) suggests that neuromuscular ageing is basically related to muscle structure remodeling. However, when the data between the two extremes of the curve are considered, alterations of neural drive are found; these may be responsible for the specific neural strategies adopted by OM when performing daily activities. It thus appears that older adults inevitably function at a higher level of muscle activation to perform submaximal tasks. This may expose them to the risk of premature fatigue during prolonged submaximal efforts. Hence, quantification of neural involvement in the execution of daily activities seems important for the identification of the factors contributing to the loss of functional mobility and the development of physical frailty in many OM.

## Author Contributions

All authors listed, have made substantial, direct and intellectual contribution to the work, and approved it for publication.

## Conflict of Interest Statement

The authors declare that the research was conducted in the absence of any commercial or financial relationships that could be construed as a potential conflict of interest.
